# Pain acceptance and illness intrusiveness in low-back pain: A longitudinal study

**DOI:** 10.3389/fpsyt.2022.925251

**Published:** 2022-08-12

**Authors:** Eszter Simoncsics, Barna Konkolý Thege, Adrienne Stauder

**Affiliations:** ^1^Doctoral School of Semmelweis University, Budapest, Hungary; ^2^Mission Medical Center, Veresegyház, Hungary; ^3^Waypoint Research Institute, Waypoint Centre for Mental Health Care, Penetanguishene, ON, Canada; ^4^Department of Psychiatry, University of Toronto, Toronto, ON, Canada; ^5^Institute of Behavioral Sciences, Semmelweis University, Budapest, Hungary

**Keywords:** low-back pain, illness intrusiveness, pain acceptance, pain-related disability, chronic pain, quality of life biopsychosocial

## Abstract

**Background:**

In chronic pain syndromes, acceptance of pain may be a better approach than pain control. So far, little data have been available on how pain and its acceptance affect illness intrusiveness among patients with low-back pain (LBP).

**Objective:**

The present longitudinal study evaluates the impact of pain acceptance on illness intrusiveness in patients with LBP.

**Methods:**

Study participants were asked to complete the following questionnaires during their visit (T1) at one of four diverse rheumatologic outpatient clinics, and then 2–3 months later (T2) *via* phone or online: Chronic Pain Acceptance Questionnaire (CPAQ), Illness Intrusiveness Rating Scale (IIRS), Roland-Morris Disability Questionnaire (RMDQ), Patient Health Questionnaire Depression subscale (PHQ9), and socioeconomic data.

**Results:**

One hundred and twenty-seven individuals completed the questionnaires at baseline (31 having acute, 15 subacute and 81 chronic low back pain) and 97 at follow-up. Illness intrusiveness was negatively correlated with chronic pain acceptance both at T1 (r = −0.39) and T2 (*r* = –0.44). Illness intrusiveness scores have not changed significantly from T1 (*M* = 28.59 SD = 13.08) to T2 (*M* = 28.24, SD = 15.76). In a multiple regression model—including pain intensity, functional status, pain acceptance, depression severity, age, sex and educational level—the independent predictors of follow-up illness intrusiveness scores were lower pain acceptance and higher depression scores.

**Conclusions:**

In our study, patients with acute, subacute and chronic low back pain reported similar levels of illness intrusiveness. In addition, illness intrusiveness scores have not changed significantly during the 2-month follow-up period and pain acceptance proved to be a significant independent predictor of illness intrusiveness among patients with chronic low-back pain.

## Introduction

Chronic low-back pain has a significant impact on everyday functioning and can lead to—among other negative psychosocial outcomes—work disability. The biopsychosocial approach of chronic pain emphasizes the importance of the evaluation of the pain experience and the related individual coping strategies. Higher distress is associated with depression (1), pain catastrophizing (2) or fear avoidance beliefs (3) resulting in more pronounced focus on pain experience and more avoidance behaviors, preventing sufferers from normal everyday activities or even physical therapy.

The concept of pain acceptance, as an alternative to an active attempt to control pain, has attracted much attention in the past decades, and recently it has been included in the guidelines as a first-line recommendation for the biopsychosocial management of chronic pain (4). Pain acceptance is an active coping strategy that redirects the focus from pain control to engagement in valued activities. Acceptance means responding to pain-related experiences without attempts to control or avoid pain; instead, individuals accepting their pain tend to tolerate the discomfort of pain to live a valued life, in accordance with personal goals (5). Many studies confirmed that the acceptance of pain results in better daily functioning, decrease in pain severity (6–8) and a better quality of life (6–10).

Thus, the goals of the (self-) management of chronic conditions in general, and of chronic low-back pain specifically, go beyond the control of the symptoms. Health-related quality of life (HRQOL) is a key indicator of the successful management of any chronic disease. It can be assessed in numerous ways, using general or disease-specific, objective or subjective indicators. The main domains of HRQOL measures are somatic symptoms, physical functionality, social activity, and psychological well-being (9). The concept of illness intrusiveness proposed by Devins emphasizes that the experienced burden is not only caused by the disease itself but medical treatment (time, schedule, costs, side effects, etc.) can also make an individual's life difficult (10). Individuals struggling with chronic disease and its treatment can be inhibited in doing their daily activities and can be prevented from participating in their social roles and familial activities. In this model, illness intrusiveness reflects the lifestyle disruptions that contribute to the deterioration of quality of life (11, 12). The theoretical framework also emphasizes that although illness intrusiveness mediates a substantial part of the negative effects of a chronic disease on subjective well-being, a number of “psychological, social and contextual factors influence subjective well-being directly, moderate the effects of disease and treatment on illness intrusiveness and moderate the effects of illness intrusiveness on subjective well-being” (9, page 72, Figure 9). Among those psychological factors, Devins highlights the importance of perceived sense of control, while strong evidence supports the close association of depressive symptoms (a potential consequence of the combination of low level of perceived control and adverse experiences) with illness intrusiveness (9).

Surprisingly, we were not able to identify any studies on the association between illness intrusiveness and pain acceptance, a psychological factor considered important in decreasing the burden of chronic pain. The similarities of the two theoretical concepts, both focusing on valued and meaningful activities—illness intrusiveness from the burden point of view, acceptance from the positive psychological point of view—inspired our study to explore the associations between them.

In our study, we used the Illness Intrusiveness Rating Scale (IIRS) developed by Devins to operationalize his theory (10, 13). The IIRS can be conveniently used in clinical settings to estimate lifestyle disruptions, to plan interventions, and to evaluate changes over time. It has been widely used among patients with various types of chronic diseases [for a review, see Devins (13)]. However, we are unaware of any studies focusing on patients with low-back pain (LBP). Therefore, the present study would be the first to assess illness intrusiveness among patients with LBP in clinical settings.

We conducted a prospective, questionnaire-based study in a clinical sample of patients suffering from LBP, which allowed us not only to gain a cross-sectional picture of illness intrusiveness in LBP, but at the same time made it possible to explore the trajectory of LBP symptoms and the predictors—with a special focus on pain acceptance—of illness intrusiveness. More specifically, the aims of this study were (1) to describe illness intrusiveness as a function of LBP illness duration and to monitor its change over time in a clinical sample of low-back pain patients; (2) to investigate the relationship between pain acceptance and illness intrusiveness in patients with LBP; and finally, (3) to estimate the impact of pain acceptance on illness intrusiveness controlling for other psychosocial and socioeconomic factors in chronic LBP patients.

## Methods

### Participants and procedure

The study protocol was approved by the Regional Ethical Committee (SE RKEB No 108/2020). Patients suffering from low-back pain and presenting for treatment at selected rheumatologic outpatient clinics were asked to participate in the study. Inclusion criteria were diagnosed low-back pain, age between 18 and 70 years, willingness to fill in the questionnaires, and agreement to participate in the follow-up. Exclusion criteria included pain caused by a specific syndrome (tumor, infection, fracture), substance addiction, and severe psychiatric disorder (psychosis). To achieve a satisfactory number of cases and to diminish the potential bias due to differences between regional and care funding systems, we selected four rheumatologic outpatient clinics in Hungary where the attending physicians agreed to participate. Two of the three participating public clinics and the private clinic are situated in the capital (Budapest), and the third public clinic involved in the study is located in a medium-size Hungarian town. The treating rheumatologists invited their patients to participate in the study, and after securing informed consent, they handed over the printed questionnaires to participants, or the link to the online version of the questionnaire was sent to the participants *via* email, based on the contact information provided on the consent form. No incentives were given either to the participants or the physicians recruiting them.

Approximately half of the questionnaires were collected at the private clinic (*N* = 62, 48.8%), and the rest (*N* = 65, 51.2%) at the three public outpatient clinics. In Hungary, public outpatient clinics are free for insured patients in contrast to private clinics where patients have to pay for the treatment and consequently, patients with higher socioeconomic status tend to use these services. Between the baseline and the follow-up assessment, participating patients received the usual care, generally a combination of pharmacotherapy (73%), physiotherapy (70%), exercise therapy at the clinic (72%) or at home (65%), and acupuncture (11%). The participants were asked to complete the follow-up questionnaires after 2 months, in line with the significance of this time period: if there is no significant improvement in low-back pain symptoms within 2 months, typically no further improvement can be expected with conventional treatments (14). To improve retention rate, each participant was approached three times at follow-up, if not responding to the first invite.

Altogether, 127 patients agreed to participate; 79 of whom completed the questionnaires in printed format, while 48 completed them online by opening the survey link sent *via* email. The follow-up test battery was completed by 97 (76.4%) of the baseline sample; 64 participants completed the assessments online and 33 *via* phone. Participants completing both assessments were slightly older than those completing the test battery only at baseline (47.0 years compared to 42.5, *p* = 0.067), while there was no statistically significant difference between the two subgroups regarding sex, level of education, employment status, pain level, functionality, pain acceptance or depression scores. Detailed description of the sample is presented in Tables 1, 2.

**Table 1:** Characteristics of the sample at recruitment [categorical variables are characterized by occurrence and percentage, while continuous variables (age and scale scores) by mean and standard deviation].

	**Acute LBP**	**Subacute LBP**	**Chronic LBP**
	***N =* 31**	***N =* 15**	***N =* 81**
Age (years)	47.08 (12.45)	45.55 (12.71)	47.13 (11.48)
**Sex**			
Male	11 (8.66)	8 (6.29)	34 (26.77)
Female	20 (15.75)	7 (5.51)	47 (37.00)
Living with partner	24 (18.90)	10 (7.87)	56 (44.09)
**Employment type**			
Blue collar	8 (6.29)	8 (6.29)	24 (18.90)
White collar	21 (16.54)	4 (3.15)	52 (40.94)
**Employment status**			
Actively working	2 (1.57)	9 (7.09)	59 (46.46)
On sick leave	6 (4.72)	4 (3.15)	8 (6.29)
Economically inactive	3 (2.36)	2 (1.57)	14 (11.02)
**Education**			
Primary school	0	3 (2.36)	5 (3.94)
Vocational training	3 (2.36)	2 (1.57)	12 (9.45)
Vocational high school	6 (4.72)	4 (3.15)	12 (9.45)
High school	6 (4.72)	1 (0.78)	14 (11.02)
University degree	16 (12.60)	5 (3.94)	38 (29.92)
**Residence**			
Capital	24 (18.90)	23 (18.11)	57 (44.88)
City	5 (3.94)	2 (1.57)	18 (14.17)
Rural	2 (1.57)	0	6 (4.72)
**Financial status**			
Lives very sparingly	0	0	1 (0.78)
Sparingly	2 (1.57)	0	5 (3.94)
Average	16 (12.60)	9 (7.08)	39 (30.71)
Good	13 (10.23)	5 (3.94)	32 (25.19)
Very good	0	1 (0.78)	4 (3.15)
Pain intensity	4.65 (1.99)	5.53 (2.87)	4.93 (2.96)
Illness intrusiveness	28.0 (10.58)	27.14 (14.26)	29.07 (13.84)
Depressive symptoms	6.78 (5.35)	8.16 (5.7)	6.76 (5.42)
Functioning / disability	9.32 (4.85)	9.73 (6.39)	8.30 (5.11)
Pain acceptance	23.00 (6.93)	25.63 (9.29)	27.81 (6.91)

**Table 2:** The mean scores of the questionnaires at baseline and at follow-up.

	***N***	**Baseline (T1)**		**Follow-up (T2)**		**Change**	***t***	***p***	**Hedges' g**
		***M***	**SD**	***M***	**SD**				
PAINmax	97	6.2	2.36	4.62	3	−1.58	5.342	<0.001	0.58
PAINmean	97	5.03	2.27	4.32	2.7	−0.71	2.714	0.008	0.28
CPAQ	87	27.39	7.15	29.82	7.8	2.43	−4.282	<0.001	−0.32
CPAQw	87	9.55	5.79	10.87	5.6	1.32	−2.286	0.025	−0.23
CPAQa	87	17.84	6.42	18.95	5.2	1.11	−2.501	0.014	−0.18
IIRS	95	28.59	13.08	28.24	15.76	−0.35	−0.151	0.88	−0.013
IIRS-instr	95	3,23	1.44	2.98	1.59	−0.25	0.48	0.632	0.04
IIRS-intim	95	2.04	1.51	1.94	1.60	−0.10	0.507	0.613	0.06
IIRS-relat	95	1.74	0.98	1.80	1.17	0.06	−0.987	0.326	−0.01
RMDQ	97	8.93	5.21	6.10	5.8	−2.83	5.45	<0.001	0.51
PHQ9	97	6.87	5.36	5.74	5.3	−1.13	2.338	0.022	0.21

PAINmax: worst pain in the last 4 days (range 0–10); PAINmean: average pain intensity in the past 4 days (range 0–10); CPAQ: Chronic Pain Acceptance Questionnaire total score; CPAQw: Chronic Pain Acceptance Questionnaire, Willingness Subscale; CPAQa: Chronic Pain Acceptance Questionnaire, Activity Subscale; IIRS: Illness Intrusiveness Rating Scale (subscales: IIRS-inst: Instrumental Domain; IIRS-intim: Intimacy Domain; IIRS-relat: Relationships and Personal Development Domain); RMDQ: Roland-Morris Disability Questionnaire; PHQ9: Patient Health Questionnaire Depression Subscale.

Hedges' g interpretation: 0.2 ≤ small effect, 0.5 ≤ medium effect, 0.8 ≤ large effect.

### Assessment strategy

#### Assessments completed both at baseline (T1) and follow-up (T2)

Actual, average, and maximum pain intensity was evaluated on a Numeric Rating Scale (NRS) from 0 (no pain) to 10 (the most intense pain possible). Further, the Hungarian version of the following validated or widely used questionnaires was also administered at both time points: Chronic Pain Acceptance Questionnaire (CPAQ), Illness Intrusiveness Rating Scale (IIRS), Roland-Morris Disability Questionnaire (RMDQ), and the Depression Subscale of the Patient Health Questionnaire [(PHQ9) (13, 15)].

#### Questionnaires administered at baseline only

Duration of pain symptoms was assessed by a single question where respondents had six answer options ranging from “ <3 days”, “4 days to 1 week”, “one to 3 weeks”, “3–6 weeks”, “6–12 weeks”, “more than 3 months”. We categorized the respondents as having acute (pain duration of 6 weeks or less), subacute (6–12 weeks duration) and chronic pain (pain duration of more than 3 months). Socioeconomic characteristics assessed included age, sex, marital status (living alone or with a partner), level of education (primary school, vocational training, vocational high school, high school and college or university degree), residence (capital, city or rural), self-assessed financial status (lives very sparingly, sparingly, average, good or very good); employment status (working full time, being on sick leave, or economically inactive) and type of work (blue- or white collar).

### Measures

#### Chronic Pain Acceptance Questionnaire

The 20-item CPAQ was developed and validated by McCracken, Vowles and Eccleston in 2004. Each statement can be scored on a Likert scale of 7 options, ranging from 0 (never true) to 6 (always true). The two subscales are Pain Willingness (CPAQ-w, 10 items) and Activity Engagement (CPAQ-a, 10 items). The former, which focuses on mental openness to discomfort, reflects on how much one accepts that she/he cannot influence or avoid pain, while the latter focuses on the physical and social behavior of being active and participating in life despite discomfort (16).

The CPAQ-20 was translated into many languages, the Hungarian version was translated and reported on in a PhD thesis (17). A shorter, eight-item version (four items for each subscale) of the CPAQ was published by Fish at al. in 2010 (18) and was proved to have good reliability and validity also in the Norwegian (19) and Chinese (20) context. In the present study, this abbreviated, 8-item version was used, the internal consistency was good in the present data set (Cronbach's alpha of 0.769 for the total score, 0.833 for CPAQ-a, and 0.781 for the CPAQ-w).

#### Illness Intrusiveness Ratings Scale

The Illness Intrusiveness Ratings Scale measures the extent to which a disease or its treatment or both interfere with activities in important life domains such as health, diet, work, active recreation, passive recreation, finances, relationships, sexual life, family, social life, self-expression, religion, and community life (10, 13, 21). In relation to each domain, the IIRS asks the question “How much does/do your illness/es and/or its treatment interfere with …”. The answer options range from 1 (not very much) to 7 (very much) for each item. Summing the answers gives a total score of 13 to 91. The questionnaire has a three-factor structure, related to “Relationships and Personal Development,” “Intimacy” and “Instrumental” life domains (13). As the number of items is different in these domains, mean values were calculated to allow direct comparability of domain scores; higher values indicate higher disease burden. The Hungarian version of the IIRS and its subscales were validated by Novak and colleagues among dialysis patients in 2005 (22). Internal consistency of the subscale and total scores in the present sample was good (Cronbach's alpha of 0.812 for Relationships, 0.770 for Intimacy, 0.728 for Instrumental, and 0.882 for the total score).

#### Patient Health Questionnaire, Depression Subscale (PHQ9)

The Depression Subscale (PHQ9) of the Patient Health Questionnaire consists of nine items. Each item is scored on a scale from 0 to 3 and indicates a typical depressive symptom, the higher number indicating higher severity of the given symptom. Based on the total score, depression severity categories are estimated as 0–4 minimal, 5–9 mild, 10–14 moderate, 15–19 moderately severe or 20–27 severe depressive syndrome (23). In the present study, the not-yet validated but publicly available (https://multiculturalmentalhealth.ca/wp-content/uploads/2019/07/PHQ-9-Hungarian.pdf; https://ifightdepression.com/hu/hangulatmero-teszt) Hungarian version was used. Internal consistency of the scale in the present sample was excellent (Cronbach's alpha = 0.882).

#### Roland-Morris Disability Questionnaire

The RMDQ is a 24-item self-report questionnaire on how low-back pain affects functional activities. Sample items include “I stay at home most of the time because of my back”, “I change position frequently to try and get my back comfortable.” Answers can be yes (1) or no (0) and total scores range from 0 (no disability) to 24 (severe disability). In our study, Cronbach's alpha was 0.864. This assessment tool is the most sensitive for patients with mild to moderate disability due to acute, sub-acute or chronic low-back pain (15, 24).

### Statistical analysis

The Statistical Package for the Social Sciences (SPSS) was used for statistical analysis. Descriptive statistics (N and % as well as M and SD) were used to characterize the sample across study variables. Given the non-significant deviation from the normal distribution in case of our continuous variables, parametric statistical methods were used to analyze their relationships. We used the paired sample *t*-test to estimate changes from baseline (T1) to follow-up (T2). Effect size was calculated with a web-based application (https://effect-size-calculator.herokuapp.com/#form3) where applicable. Pearson correlation was used to analyze the cross-sectional relationship between the continuous variables. We conducted hierarchical multiple linear regression to investigate the predictive power of predictors on illness intrusiveness at follow-up. In the regression analysis, only patients who had any pain at follow-up were included (as no illness intrusiveness was assumed in the absence of pain). *P*-values below or equal to 0.05 were considered as statistically significant. Considering the potential overlap between some of the independent variables, multicollinearity was examined throughout the regression models. As tolerance values exceeded 0.41 in each case (suggested lower limit: 0.40) and variance inflation factor values did not exceed 2.46 (suggested upper limit: 5.00), we concluded that multicollinearity did not pose a serious threat to the reliability of our regression models.

## Results

### Illness duration and illness intrusiveness profiles

**Figure 1:**
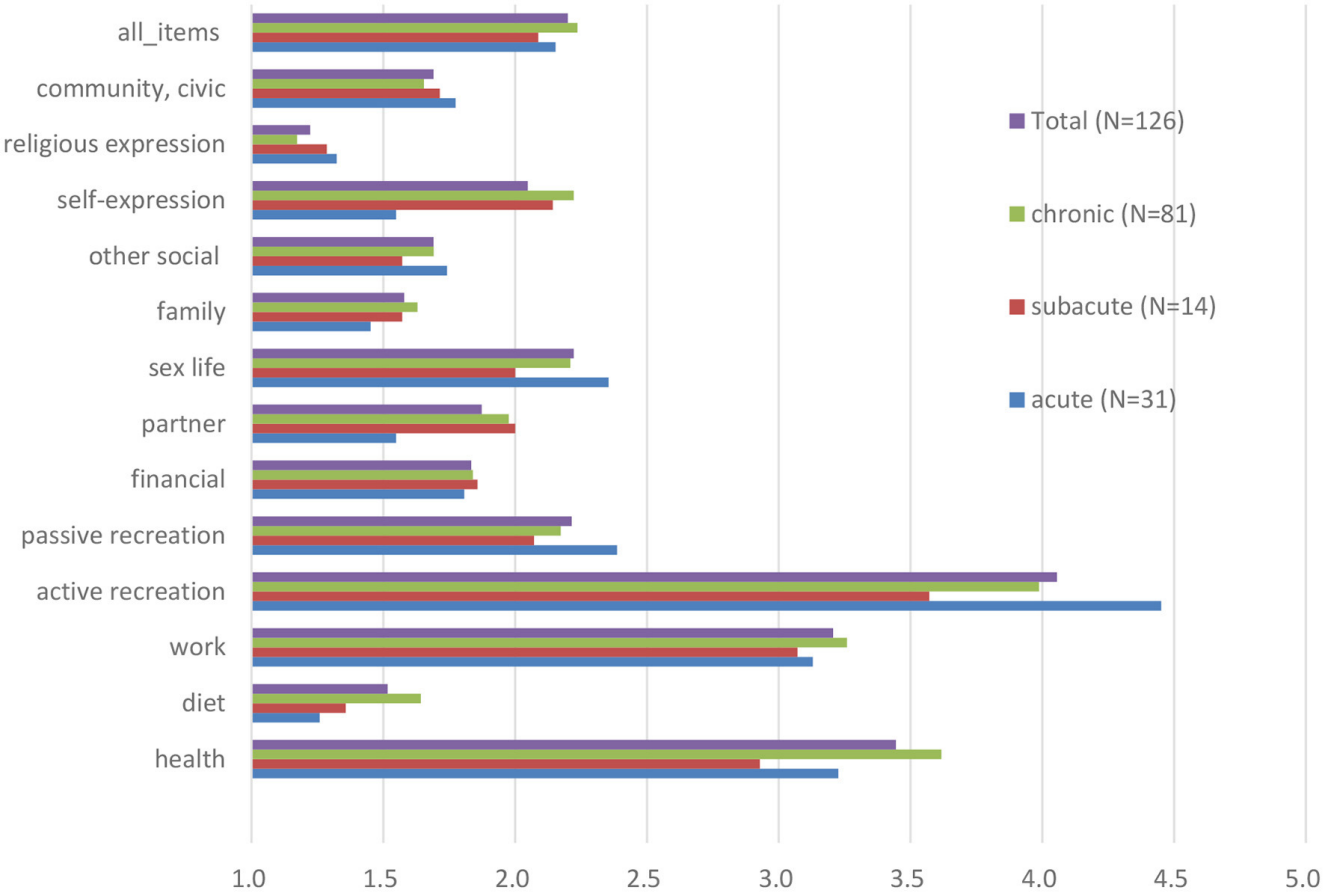
Illness Intrusiveness Ratings Scale scores (mean of all items and individually for each item) among patients with low back pain, stratified by pain duration at baseline.

We formed three subgroups according to the duration of LBP: acute LBP (31 participants), subacute LBP (15 participants), and chronic LBP (81 participants). The comparison of the three groups according to illness intrusiveness can be found in Figure 1. We found no statistically significant difference among the subgroups regarding any of the IIRS items or their pooled mean. The three areas where the burden of LBP was most prominent in the total sample were active recreation (3.97 ± 2.33), health (3.33 ± 1.91), and work (3.07 ± 2.12).

### Changes over time

Pain intensity scores, functional level and psychological characteristics of the sample at baseline and follow-up are summarized in Table 2. Although overall pain intensity has decreased, this improvement was significant only for the acute pain subgroup, where the mean pain intensity decreased from 4.92 (±1.98) at baseline to 1.96 (±1.92) at follow-up (*t* =4.864, *p* < 0.001, *d* = 1.46). The change in the group with subacute (score change from 5.09 ± 2.51 to 3.27 ± 2.76) and chronic (score change from 4.98 ± 2.78 and 4.25 ± 2.38) pain was not significant. Functionality, assessed by the Roland-Morris Disability Questionnaire, significantly improved over time. Pain acceptance also significantly increased but only to a small degree. Depressive symptom scores showed a small but statistically significant decrease. Interestingly, illness intrusiveness mean scores did not change either in case of the total score or in terms of its three subdomains.

### Bivariate associations between illness intrusiveness and other scales

The results confirmed the existence of a significant relationship between illness intrusiveness, somatic symptoms, and the psychological factors assessed. The correlation coefficients presented in Table 3 illustrate that IIRS scores at follow-up (IIRS_T2), as well as its subscales were similarly correlated with the predictor variables measured at the two time points, with stronger relationships at follow-up. The cross-sectional correlation of follow-up illness intrusiveness scores was moderately strong with pain intensity (*r* = 0.34) and pain acceptance (*r* = −0.441), while strong with depressive symptoms (*r* = 0.546) and disability (*r* = 0.506). The strongest correlation was found between the IIRS Instrumental Subscale and disability assessed by the RMDQ (*r* = 0.567), both reflecting poor physical functionality. Interestingly, while the Willingness Subscale of the CPAQ was moderately correlated, the Activity Subscale was not significantly correlated with any of the IIRS scores. Pain intensity was more strongly correlated with the IIRS Instrumental Subscale than the Relational Subscale and not significantly related to the Intimacy Subscale of the IIRS. Although for the whole sample there was no significant change in IIRS scores over time, the changes in IIRS total score inversely correlated with the changes in CPAQ total scores (*r* = –0.291, *p* < 0.01), indicating that increased pain acceptance was related to decreased illness intrusiveness.

**Table 3:** Correlation between follow-up (T2) illness intrusiveness and the measures of pain, functionality, and psychological factors at baseline (T1) and follow-up (T2).

	**IIRS_T2**	**IIRS.INST_T2**	**IIRS.INTIM_T2**	**IIRS.RELAT_T2**
IIRS_T1	0.628^**^	0.521^**^	0.542^**^	0.615^**^
RMDQ_T1	0.427^**^	0.422^**^	0.267^**^	0.391^**^
RMDQ_T2	0.506^**^	0.567^**^	0.284^**^	0.416^**^
PHQ9_T1	0.385^**^	0.343^**^	0.342^**^	0.296^**^
PHQ9_T2	0.546^**^	0.514^**^	0.394^**^	0.498^**^
Painmean_T1	0.281^**^	0.335^**^	0.176	0.157
Painmean_T2	0.344^**^	0.444^**^	0.154	0.226^*^
Painmax_T1	0.162	0.213^*^	0.082	0.071
Painmax_T2	0.360^**^	0.455^**^	0.168	0.238^*^
CPAQ_T1	−0.311^**^	−0.269^*^	−0.255^*^	−0.322^**^
CPAQ_T2	−0.441^**^	−0.482^**^	−0.282^**^	−0.369^**^
CPAQa_T1	−0.034	0.028	−0.017	−0.111
CPAQa_T2	−0.172	−0.17	−0.081	−0.193
CPAQw_T1	−0.336^**^	−0.351^**^	−0.287^**^	−0.269^*^
CPAQw_T2	−0.452^**^	−0.508^**^	−0.314^**^	−0.336^**^

IIRS: Illness Intrusiveness Rating Scale (subscales: IIRS-inst: Instrumental Domain; IIRS-intim: Intimacy Domain; IIRS-relat: Relationships and Personal Development Domain), RMDQ: Roland-Morris Disability Questionnaire; PHQ9: Patient Health Questionnaire Depression Subscale; PAINmean: average pain intensity in the past 4 days; PAINmax: worst pain in the last 4 days; CPAQ: Chronic Pain Acceptance Questionnaire total score; CPAQa: Chronic Pain Acceptance Questionnaire, Activity Subscale; CPAQw: Chronic Pain Acceptance Questionnaire, Willingness Subscale.

* p ≤ 0.05.

** p ≤ 0.01.

### Multivariate analyses

In the multivariate analyses (Table 4), we tested both the cross-sectional and longitudinal relationship of pain acceptance with T2 illness intrusiveness controlling for other psychosocial and demographic variables known to be associated with illness intrusiveness. In these hierarchical regression models, we only included patients who reported any pain at follow-up (*N* = 89).

**Table 4:** Biopsychosocial predictors (assessed at baseline in Model 1 vs. at follow up in Model 2) of follow-up illness intrusiveness in patients with chronic low-back pain (hierarchical multiple regression).

	**Predictors**	**Standardized beta**	***t***	***R* ^2^**	***R*^2^ change**	***F* change**
**Model 1**						
Step 1				0.148	0.148	12.503^***^
	CPAQ_T1	−0.385	−3.536^***^			
Step 2				0.208	0.060	1.738
	CPAQ_T1	−0.360	−3.211^**^			
	Age	−0.033	−0.290			
	Sex	−0.192	−1.762			
	Education	−0.190	−1.718			
Step 3				0.263	0.055	5.114^*^
	CPAQ_T1	−0.369	−3.390^***^			
	Age	−0.036	−0.328			
	Sex	−0.081	−0.692			
	Education	−0.109	−0.966			
	Painmean_T1	0.267	2.261^*^			
Step 4				0.289	0.026	2.418
	CPAQ_T1	−0.292	−2.459^*^			
	Age	−0.064	−0.581			
	Sex	−0.062	−0.539			
	Education	−0.053	−0.447			
	Painmean_T1	0.167	1.249			
	RMDQ_T1	0.231	1.555			
Step 5				0.354	0.066	6.704^**^
	CPAQ_T1	−0.316	−2.763^**^			
	Age	−0.046	−0.436			
	Sex	−0.015	−0.130			
	Education	−0.049	−0.436			
	Painmean_T1	0.168	1.313			
	RMDQ_T1	0.074	0.476			
	PHQ9_T1	0.304	2.589^**^			
**Model 2**						
Step 1				0.255	0.255	27.713^***^
	CPAQ_T2	−0.505	−5.264^***^			
Step 2				0.320	0.065	2.500
	CPAQ_T2	−0.499	−5.147^***^			
	Age	−0.056	−0.578			
	Sex	−0.215	−2.278^*^			
	Education	−0.176	−1.825			
Step 3				0.373	0.052	6.419^*^
	CPAQ_T2	−0.492	−5.245^***^			
	Age	−0.100	−1.045			
	Sex	−0.171	−1.835			
	Education	−0.129	−1.359			
	Painmean_T2	0.242	2.533^*^			
Step 4				0.405	0.032	4.087^*^
	CPAQ_T2	−0.406	−4.017^***^			
	Age	−0.124	−1.316			
	Sex	−0.161	−1.764			
	Education	−0.041	−0.401			
	Painmean_T2	0.135	1.250			
	RMDQ_T2	0.264	2.022^*^			
Step 5				0.488	0.084	12.258^***^
	CPAQ_T2	−0.334	−3.450^***^			
	Age	−0.133	−1.511			
	Sex	−0.100	−1.146			
	Education	−0.007	−0.075			
	Painmean_T2	0.115	1.143			
	RMDQ_T2	0.155	1.228			
	PHQ9_T2	0.348	3.501^***^			

Painmean: average pain intensity in the past 4 days; RMDQ: Roland-Morris Disability Questionnaire; CPAQ: Chronic Pain Acceptance Questionnaire; PHQ9: Patient Health Questionnaire Depression Subscale; T1: variable assessed at baseline; T2: variable assessed at follow-up.

* p ≤ 0.05.

** p ≤ 0.01.

*** p ≤ 0.001.

In Model 1, we tested the independent predictive role of baseline (T1) pain acceptance scores on illness intrusiveness at follow-up, entering the variables hierarchically (Step 1: pain acceptance; Step 2: sociodemographic variables: age, sex, education; Step 3: pain intensity; Step 4: disability; Step 5: depressive symptoms); see Table 4, Model 1. The results showed a moderately strong, negative association between pain acceptance and illness intrusiveness (beta = −0.385, *p* = 0.001), that did not change significantly after entering the sociodemographic variables into the model. Pain intensity itself (step 3) revealed to be a weak, independent predictor of illness intrusiveness (beta = 0.267, *p* = 0.027) without considerably influencing the association between pain acceptance and illness intrusiveness. When entering disability into the model at step 4, the predictive role of pain acceptance in relation to illness intrusiveness decreased but it did not become insignificant. Finally, in step 5, depression severity was entered into the model. This final model explained 35.4% of the variance in follow-up illness intrusiveness scores, and confirmed that baseline pain acceptance (beta = −0.316, *p* = 0.007) and baseline depressive symptoms (beta = 0.304, *p* = 0.012) were both moderately strong predictors of illness intrusiveness at follow-up, independently from pain intensity, disability, sociodemographic factors, and each other.

In Model 2 (Table 4), we investigated the same variables but in a cross-sectional design: all variable scores were based on the follow-up survey (T2). Pain acceptance in this model was more strongly correlated with illness intrusiveness (beta = −0.505, *p* < 0.001). At step 2, among the sociodemographic variables, only being male was a significant predictor of illness intrusiveness (beta = 0.215, *p* = 0.025) but entering these variables into the model did not change the predicative power of pain acceptance considerably. At step 3, when pain intensity was entered, this variable was significantly associated with illness intrusiveness (beta = 0.242, *p* = 0.013) but changed the predictive role of pain acceptance only in a negligible way. Similar to the longitudinal model, entering disability level at step 4 revealed that while this variable was a significant predictor of illness intrusiveness (beta = 0.264, *p* = 0.047), it decreased the predictive power of pain acceptance only moderately. As we entered depressive symptomatology into the model at step 5, the predictive value of pain intensity and disability became non-significant, while depressive symptoms proved to be a similarly strong predictor of illness intrusiveness as pain acceptance, both having moderately strong independent association with the dependent variable (betas of 0.348 and −0.334, *p* = 0.001, respectively). The final model explained 48.8% of the variance in illness intrusiveness scores.

## Discussion

### Interpretation of the results

In our study, we evaluated illness intrusiveness in a clinical sample of patients with LBP. We investigated the impact of pain acceptance on illness intrusiveness controlling for other psychosocial factors known to influence patients' everyday life experience and relationships. The importance and current relevance of this research is underlined by the recent inclusion of the concept of acceptance as a therapeutic focus in guidelines for chronic pain management [e.g., NICE guidelines for chronic pain (25)]; however, this approach has not yet been universally implemented in everyday clinical practice.

Illness intrusiveness scores (assessed by the IIRS) in the present sample (28.59 ± 13.08 at baseline, 28,24 ± 15.76 at follow-up) were considerably lower than those reported in previous studies for other pain syndromes such as fibromyalgia (54.8 ± 17.24), osteoarthritis (42.2 ± 18.75) (13) or traumatic brachial plexus injury (IIRS 40.0 ± 18.0) (26). A potential explanation for this discrepancy may be the fact that our sample was recruited at general rheumatology clinics instead of specialized pain clinics. Worthy of note though that the mean IIRS score of our clinical sample was slightly higher than that (19.00 ± 16.26) of 1,575 persons reporting musculoskeletal symptoms in a Hungarian representative health survey but lower than the IIRS scores reported by people with psychiatric diseases or cancer in the same Hungarian survey including 12,700 respondents (27).

The areas of everyday life most impacted by low-back pain besides health were work and active recreation. While it was anticipated that a musculoskeletal symptom would considerably impact movement and physical activities, it was more surprising that overall illness intrusiveness did not significantly change during the two-month follow-up period despite the fact that pain decreased to some extent and functional level improved significantly. Our results also revealed that pain intensity only moderately correlated with illness intrusiveness, while depressive symptoms and pain acceptance showed a stronger association with this construct similar to other studies (28).

The correlation between illness intrusiveness and disability attributed to LBP was also pronounced. The same tendency has also been found in other studies; for instance, Novak and colleagues found a stronger correlation (*r* = 0.738) between pain-related disability and illness intrusiveness than pain intensity itself and illness intrusiveness (*r* = 0.500) in patients with peripheral nerve injury (26). When considering the IIRS subscales, intimacy is less impacted by pain and more by depressive symptoms than the instrumental areas. These findings confirm that specific somatic symptoms are less closely related to perceived illness intrusiveness than general functional level and psychosocial wellbeing.

The results of the present study give support to our hypothesis that pain acceptance is negatively associated with illness intrusiveness, thus it may serve as a protective factor. Bivariate analyses revealed a moderately strong correlation between illness intrusiveness and pain acceptance, which was very similar at both time points (*r* = −0.444 at T1 and −0.431 at T2). Further analysis of this relationship revealed that pain acceptance correlated with all subdomains of IIRS (Instrumental as well as the Social and Intimacy Subscale).

An unexpected result of our analysis was that while the Willingness Subscale of the Chronic Pain Acceptance Questionnaire was strongly correlated with illness intrusiveness, especially regarding the Instrumental Subscale, the Activity Subscale was not significantly correlated with any of the IIRS domains either at T1 or T2. This somewhat counterintuitive finding might be explained by the fact that routine care emphasizes the importance of physical activity but does not deal with the psychological challenges of living with pain. Scores on the Willingness Subscale of the Chronic Pain Acceptance Questionnaire were significantly lower than those on the Activity Subscale suggesting that patients with low-back pain tend to stay physically active more easily despite chronic pain than accept the perspective that their pain will remain/become chronic.

The multivariate analyses confirmed the results of the bivariate analyses where the moderately strong correlation between pain acceptance and illness intrusiveness proved to be independent from other already known predictors. With regards to the sociodemographic factors, we could observe the expected trends (lower age, male sex and lower level of education were associated with higher illness intrusiveness scores), but overall they have not reached the level of significance. The disease-related indicators (pain intensity and disability / functional level) explained a smaller proportion of the variance than the psychological factors: pain acceptance and depressive symptoms improved the explanatory power of the model to a large extent. The cross-sectional associations measured at follow-up could be predicted by the longitudinal regression model, the baseline characteristics explained 35.4% of the variance in follow-up illness intrusiveness scores. These findings are consistent with those of Kapadi and colleagues who concluded that pain acceptance was the main independent predictor of physical and psychological quality of life among women with primary dysmenorrhea (29).

Our results confirm the importance of subjective reactions to pain beyond pain intensity itself in the context of illness intrusiveness in low back pain. These results are comparable to other studies indicating that pain acceptance is the key determinant of quality of life among patients with chronic low-back pain. The study of McCracken and colleagues, including 160 patients seeking treatment for chronic pain (30), concluded that greater acceptance of pain was associated with lower reported levels of pain, less pain-related anxiety and avoidance, less depression and disability, and better employment status. In a similar study, the significant relationships between pain acceptance and functioning were independent of pain intensity (31).

From a practical point of view, our follow-up study supports the notion that the assessment of pain acceptance during routine medical encounters can predict mid-term outcomes in terms of illness intrusiveness. Our findings may contribute to the more extensive clinical implementation of the biopsychosocial approach as recommended by current chronic pain management guidelines (25), which emphasize that therapeutic interventions should not focus only on the somatic changes or the decrease of pain intensity but on functional level and quality of life as well reflected by the daily activities connected to personal goals and values. This active, desirable aspect of pain acceptance is important to differentiate from passive acceptance, which would imply simply giving up hope for a life worth living. The theoretical basis for the mechanism of active pain acceptance is psychological flexibility (6), which captures how effectively an individual can mobilize his or her own internal resources in a complex way for the sake of a valuable goal (18).

Acceptance and Commitment Therapy (ACT) has adopted this approach and accordingly, one of its major therapeutic goals is the increase of psychological flexibility, together with improving the tolerance for negative emotions (32). It is not surprising therefore that the clinical efficacy of ACT in chronic pain has been confirmed by a number of studies, the effect sizes ranging from small to medium for most outcome measures such as physical functioning, pain acceptance, anxiety or depression (33). However, intervention effects of ACT on pain intensity and quality of life were small in an earlier (34) and not significant according to a more recent meta-analysis (35). Worthy of note though that these results might be influenced by the outcome measures used for quality of life (36). For example, a general quality of life measure such as the SF-36 might be less sensitive to changes in acceptance of pain than the Illness Intrusiveness Scale, which focuses specifically on the perceived limitations caused by the disease. In everyday practice, Lin and colleagues examined the effectiveness of an Internet-based ACT intervention. Participants reported less pain and a better quality of life at the end of the therapy and after 12 months (37). Although the IIRS has not been used in any ACT efficacy study yet, based on our results, employing illness intrusiveness as an outcome variable in ACT efficacy/effectiveness research seems to be a promising direction. From a clinical perspective, an important advantage of the constructs of illness intrusiveness and pain acceptance is that relatively brief and effective assessment tools are available to measure these variables so they can be easily included in routine clinical care.

### Strengths of the present study

The longitudinal data collection allowed us to test the cross-sectional relationships at two time points, and we could also test the predictive value of biopsychosocial indicators at the time of the first clinic visit on mid-term outcomes. Patients were recruited at four typical centers for rheumatologic disorders, including state financed and private practices, which increases the generalizability of the findings.

The participants of the present study represent the general low-back pain population of working age, including acute, subacute and chronic LBP cases, according to a typical distribution of patients presenting for treatment. Also, the sample included nearly an equal proportion of females and males suggesting representativity regarding sex.

### Limitations of the study

Although the sample size was fair and there was an effort to include all consecutive patients showing up for treatment in the data collection period, the present one remained a convenience sample where the number of acute and subacute patients was relatively low. The relatively small sample also resulted in a suboptimal cases to independent variables ratio in the multivariate analyses. Another limitation is that we have no exact data on refusal rate, limiting the generalizability of the results from this perspective. A further potential bias might be caused by the different ways of completing the questionnaires (paper-and-pencil, online or via a phone interview). We assume though that this bias might be small, as several studies found equivalence between paper-and-pencil versus the electronic administration of patient reported outcome measures (38). A final limitation of the present study is that a comprehensive psychometric assessment of the Hungarian version of the PHQ9 has not yet been conducted; and therefore, the psychometric adequacy of this tool is uncertain.

### Clinical implications

Acceptance-based therapies emphasize the importance of living a value-oriented life, that is, they encourage individuals to focus on goals of personal importance despite a disability, in the present case, despite chronic pain. The concept of illness intrusiveness is in line with this approach as it measures the impact of an illness on the most important areas of everyday life. Therefore, illness intrusiveness may be a practical indicator when monitoring the effectiveness of acceptance-based rehabilitation programs designed to support clients with chronic LBP.

Our results also support the importance of increasing and monitoring pain acceptance in the context of the complex biopsychosocial treatment of LBP. These data also provide additional support to the usefulness of two simple assessment tools (Illness Intrusiveness Rating Scale and the Chronic Pain Acceptance Questionnaire), which can help plan and evaluate psychosocial interventions focusing on pain acceptance and the minimization of disruptions in daily life as a result of LBP.

## Data availability statement

The raw data supporting the conclusions of this article will be made available by the authors, without undue reservation.

## Ethics statement

The study protocol was approved by the Regional Ethical Committee (SE RKEB No 108/2020). The patients/participants provided their written informed consent to participate in this study.

## Author contributions

ES and AS contributed to the conception and design of the study and organized the database. ES, BK, and AS performed the statistical analysis and wrote the manuscript. All authors contributed to manuscript revision, read, and approved the submitted version.

## Conflict of interest

The authors declare that the research was conducted in the absence of any commercial or financial relationships that could be construed as a potential conflict of interest.

## Publisher's note

All claims expressed in this article are solely those of the authors and do not necessarily represent those of their affiliated organizations, or those of the publisher, the editors and the reviewers. Any product that may be evaluated in this article, or claim that may be made by its manufacturer, is not guaranteed or endorsed by the publisher.
